# Role-based responsibilities in securing research integrity: increasing support for multi-level implementers

**DOI:** 10.3389/frma.2023.1256426

**Published:** 2023-09-14

**Authors:** Kristi Lõuk

**Affiliations:** Centre for Ethics, University of Tartu, Tartu, Estonia

**Keywords:** research integrity, roles, responsibilities, implementers, policies, procedures, research culture, research environment

## Abstract

This perspective article focuses on how researchers help to secure the research integrity-related responsibilities of various institutions in their various roles, as same researcher can fulfill the tasks of different stakeholders, be in different roles, and wear multiple hats simultaneously while performing duties at different levels. Institutions whose research integrity-related responsibilities are carried out by researchers should support the multi-level implementers more in carrying out these roles and responsibilities and consider their input when setting up tasks. In addition to having necessary policies and procedures, these should be actionable and supported by responsible research culture and environment. Furthermore, policies and action plans should be created in consideration of and in co-creation with the actual implementers. Realizing that the multiple-level role of researchers also helps to secure research integrity at the institutional and system level of science should go hand in hand with creating responsible research culture and environment where that input is taken into account. This in turn will help researchers deal with various current pressures, e.g., having not enough time or being subject to unfair evaluations.

## 1. Introduction

Research integrity (RI) as a field emerged as a response to various scandals and misconduct cases. RI pertains to conducting research in a way that ensures that it is done ethically and is trustworthy. It entails following certain principles and practices, which should be endorsed and fostered by research organizations and other stakeholders. Therefore, cases of bad practice should be noticed, as they threaten the integrity of research and undermine trust in science. In a recent meta-analysis, Xie et al. (2021) showed the prevalence of all forms of research misconduct and questionable research practices within the current environment.

The various scandals have led to agreeing on and writing down basic principles and values (e.g., in different codes and guidelines) that all researchers and other stakeholders should follow. Unlike the previous (2017) version of the European Code of Conduct for Research Integrity, which brought out for main principles, reliability, honesty, respect, and accountability, in the newly revised version (2023), the list of principles is no longer exhaustive. The latter is more in line with the picture of overall RI guidance, which has been described by Peels et al. (2019) as the existence of plurality of values that brings along a plurality of norms.

Not only are the codes and guidelines addressed to individual researchers and research teams, support staff, academies, and learned societies, but also to various other stakeholders who have roles and responsibilities in securing research integrity. The European Code also mentions research-performing organizations, funding agencies, editors and publishers. Furthermore, this list is kept open as the term “other relevant bodies” is used in the document's preamble. The preamble of the German Code (German Research Foundation, 2019/2022) specifies that complainants and ombudspersons also have the task of safeguarding good research practices.

The focus of the scholarly debate has recently been on various bearers of integrity, from research findings to science as a social system (e.g., Anderson et al., 2013; Meriste et al., 2016; Helgesson and Bülow, 2021), different stakeholders and the system of RI (Roje et al., 2022), responsible climate (Haven et al., 2020) and responsible practices (Gopalakrishna et al., 2022). Nevertheless, it seems that there is an aspect that has not gained enough attention, namely the realization of how much researchers help the institution and the scientific system carry out their research integrity-related tasks, bringing into focus the aspect of researchers as multi-level implementers. Together with making institutional policies and plans actionable and creating and maintaining a supportive research environment and culture, this will help to ease some of the current pressures researchers are currently facing.

This article begins by showing how researchers are multi-level implementers and should be supported at the institutional and system levels when carrying out role-based duties that enable the institutions to fulfill their RI-related obligations. This is followed by discussing why it is not enough to have guidelines, policies, and plans, and why these should be actionable and livable and supported by a responsible research culture and environment.

## 2. Researchers as multi-level implementers

Being a researcher does not only mean doing excellent research. Obligations extend to the research community, research participants, commissioners, funders, and collaborators to the dissemination of research results (NESH, 2022). Therefore, one should secure research grants, publish in high-quality journals with a high impact factor, lead, and act by example, treat participants with respect, help graduate students to launch a successful career, give engaging lectures, write peer reviews, perform expert evaluations, solve societal problems, and communicate research results to the wider public. The extent of engagement in these activities might vary as one can choose whether or not to take up a review for a manuscript or evaluate research projects, for example, for the European Commission. However, there is not a similar amount of autonomy after agreeing to be an RI counselor, a committee member, or a supervisor. Whereas all those mentioned above can (to some extent) be contractual duties of a researcher, they might play out at various institutional levels. Being a RI counselor for an institute or a faculty helps the institution with its RI-related tasks; being a peer reviewer or journal editor helps journals and publishers carry out their RI-related duties, being a member of an ethics or integrity committee constitutes being part of the governance structure either at the institutional or national level and thereby helps to carry out their RI related obligations. Therefore, researchers help not only the research-performing organization where they are employed, but also funding agencies, editors, and publishers to carry out their institutional RI-related duties.

However, simultaneously fulfilling all these roles and expectations is often impossible. A conflict of obligations is said to be a threat that can lead to violating moral obligations (Werhane and Doering, 1997). For example, a successful researcher who sits on various committees (institutional or nation level obligation) or is involved in public debate as an expert (social obligation) has little time for leading his/her team and supervising graduate students (obligations toward peers and junior colleagues). Fortunately, there are also a lot of guidance materials for researchers and institutions for building the necessary competencies and support (e.g., Pizzolato et al., 2022; SOPs4RI, 2023).

With all the assignments, the question arises to what extent do they constitute regular duties or should be performed on top of these? Situations where different obligations conflict could hinder the spread of a responsible research culture and environment, as one is not leading by example, which in turn may threaten research integrity, potentially leading to bad practices (e.g., self-plagiarism) or even misconduct. The researcher must be aware of these dangers and try to keep the obligations in balance. Therefore, the dilemma of practicing and making choices that are good for science but not suitable for the perspective of concrete researchers (Bouter, 2023) is not new. Solving this dilemma is not in the hands of researchers but requires policy-level changes.

One the one hand, it is up to the researcher to realize various dangers and balance his/her obligations. On the other hand, the institution should help researchers first in education and training to obtain the necessary ethical sensitivity; secondly to keep the training up to date with current challenges (e.g., use of AI, ChatGPT); thirdly, the policies should have been adopted together with the actual implements by having discussions about what should be reasonable duties for researchers at the institutional level. For example, would taking up the task of RI counselor, be seen as an administrative or research-related duty? Should one do this in addition to all already exiting obligations, or would it be possible to give up some teaching or (other) administrative tasks to clear a reasonable amount of time for this?

Although many of these issues are indeed connected to the institution and system level, as already shown, researchers are those who mostly carry out the activities. This should be adequately acknowledged. For example, it is one thing to have peer-review policies and guidelines [see COPE Council (2017) and journals]. However, as long as machines do not do peer review, the implementers of these RI practices and guidelines are primarily researchers who do this as one of their research-related tasks or on top of all their other roles and responsibilities.

## 3. Making guidelines, policies, and action plans livable

Often enough, the responsibilities stated in codes and guidelines regarding bearers of responsibility remain abstract, stating what “research institutions” should do. For example, “research institutions should” is used in the Singapore statement (Singapore Statement, 2010), and “research institutions and organizations ensure, provide” are found in the ALLEA—All European Academies (2023). Before the revised edition of the European Code, the Danish Ministry of Higher Education Science (2014) specified the bearers of responsibility. These documents outline the following bearers of research integrity: researchers, institutions, research leaders, and supervisors. As in the European Code, the Danish Code does not specify whose concrete responsibility in the institution RI is. Things are clearer when the responsibility resides in supervisors or leaders of a research team. However, institutions vary in aspects such as size and tasks, and in themselves, the codes can hardly give ready-made solutions for all.

To tackle the issue of the vagueness of guidance documents, additional materials have been issued, starting from guidance documents like the Bonn PRINTEGER Statement (Forsberg et al., 2018), where some of the bearers and implementers of the responsibilities of research-performing organizations are brought out. At the same time, the advice in this document remains rather general.

It remains unspecified who should tailor the training to suit the needs of the institution, for example, the top manager, the research integrity officer, research team leader, or someone else. The solutions may differ, but the concrete implementers must be known and agreed upon.

A step further has been made within an EU-funded project, SOPs4RI (2023), which created a toolbox where guidance materials (either already existing ones or new ones worked out by the project) can be found (e. g., building and leading an effective team, community building for a positive research culture, responsible supervision, managing competition and publication pressure). Additionally, research integrity promotion plans (RIPP) for research-performing organizations and funding organizations were worked out together with a guideline on how to create and implement a RIPP (Horbach et al., 2022; SOPs4RI, 2022). An essential part of this plan is to specify actions taken by specific people. In a RIPP, there are nine areas for improving integrity: research environment, supervision and mentoring, research integrity training, research ethics structures, dealing with breaches of research integrity, data management, research collaboration, declaration of interests and publication and communication. The plan should be a living document covering all the phases, from preparation to implementation and monitoring, and the process should be repeated occasionally. This aligns with McIntosh et al. (2023), who specify that action plans need to be complex and consider several factors aimed at changing behaviors and practices. The dynamic plan needs to cover individual, group, and contextual level causes.

With every new policy or plan, it should be kept in mind that only some of the staff may be aware of it or know the content of these documents. Therefore, the guidance documents and action plans should also be viable for successful implementation, and these should translate into practice and be accepted and followed by members of the institution.

This aligns with Degn (2020), who emphasized the need for a mediating layer between policies and individual researchers. This layer can be seen in culture, environment, and mediators. Not only leaders and managers can and should act as mediators, but the role can also be carried out by RI counselors, advisors, ambassadors, or champions. Mejlgaard et al. (2020) have shared several good examples of what institutions can do to improve their RI, whether counseling, couches, or collegiality.

Haven et al. (2020) researched the characteristics of a responsible research climate and found that fair evaluation, openness, sufficient time, integrity, trust, and freedom are essential. At the same time, lack of support, unfair evaluation policies, normalizations of overwork, and insufficient supervision of early career researchers were seen as barriers. They also looked at possible solutions, namely improving support, discussing expectations, and improving the quality of supervision. It should be noted that the offered solutions do not require an additional policy or guidance document, but they could benefit from various recommendations worked out (see e.g., Pizzolato et al., 2022; SOPs4RI, 2023). Therefore, an open environment and forum for exchange is needed. The final outcome regarding suggestions and solutions may differ as the circumstances of institutions and researchers vary. At the same time, realizing the multi-level role of researchers can be a helpful insight for further discussion on support and expectations.

## 4. Discussion

This perspective article looked at researchers' roles and responsibilities, and how these play out at different levels, from individual to institutional and at the system level. This brought out the role of researchers as double-burdened multi-level implementers. The debate has thus far mainly been concerned with the creation of necessary policies and plans, but it may be questioned whether this way, (one of) the root cause(s) is really tackled. It should be realized that whenever talking about institutional or system/national/policy level RI-related obligations, this topic should go hand in hand with creating a supportive research environment where policies and plans and actionable and livable together with the analysis of who the actual implementers could and should be and how this additional role will impact the implementer's other duties. Otherwise, there will be no foreseeable end to the debate about pressures like unfair evaluations or normalizations of overwork. Realizing the actual implementers of RI could help bearers of RI-related responsibilities at institutional and system levels construe, together with researchers, better ways of dealing with current pressures. Discussions with the participation of researchers on what constitutes overwork and what amount of working time one should have for narrow research-related activities and what for other tasks, be that teaching, mentoring, sitting in a committee (ethics, integrity), reviewing, etc., cannot be escaped and should be started as soon as possible. Not only do we want institutions and (national) policy levels who take RI responsibilities seriously, furthermore, this should be accomplished by adequately understanding and valuing the input from the actual implementers, the researchers.

## Data availability statement

The original contributions presented in the study are included in the article, further inquiries can be directed to the corresponding author.

## Author contributions

The author confirms being the sole contributor of this work and has approved it for publication.
